# Advancements in Polysaccharide-Based Nanoparticles for the Treatment of Breast Cancer: A Comprehensive Review

**DOI:** 10.3390/ph18111712

**Published:** 2025-11-11

**Authors:** Sourav Mohanto, Benachakal Honnegowda Jaswanth Gowda, Umme Hani, Soumya Narayana, Mohammed Gulzar Ahmed, Farhat Fatima, Karthika Paul

**Affiliations:** 1 Department of Pharmaceutics, Yenepoya Pharmacy College & Research Centre, Yenepoya (Deemed to Be University), Mangalore 575 018, Karnataka, India; mohanto111@gmail.com (S.M.); kalikollur123@gmail.com (S.N.); 2 Department of Pharmaceutics, College of Pharmacy, King Khalid University, Abha 61421, Saudi Arabia; uahmed@kku.edu.sa; 3 Department of Pharmaceutics, College of Pharmacy, Prince Sattam Bin Abdulaziz University, Al-Kharj 11942, Saudi Arabia; f.soherwardi@psau.edu.sa; 4 Department of Pharmaceutical Chemistry, JSS College of Pharmacy, JSS Academy of Higher Education and Research (JSSAHER), Mysuru 570 015, Karnataka, India; karthikananjundan17@gmail.com

**Keywords:** polysaccharide nanoparticles, breast cancer, triple-negative breast cancer, drug delivery, targeted therapy, chitosan, alginate

## Abstract

Breast cancer is a significant global health challenge, with rising incidence rates and substantial morbidity and mortality worldwide. Conventional treatments, while effective, often lead to adverse effects and may not fully eradicate cancer cells, resulting in recurrence and progression of tumors. Addressing these challenges requires innovative treatment strategies. Nanotechnology, particularly polysaccharide-based nanoparticles (NPs), offers a promising approach due to their biocompatibility, tunable properties, and targeted drug delivery capabilities. Polysaccharide NPs, including starch, alginate, hyaluronic acid, and chitosan, possess inherent biocompatibility and can be tailored for specific applications. Furthermore, beyond their inherent biocompatibility, polysaccharide-based NPs shown substantial interest due to their natural abundance, ease of processing, and availability from renewable resources, solidifying their role as a sustainable choice for diverse biomedical applications. By functionalizing their surface with ligands, polysaccharide NPs can target breast cancer cells, enhance therapeutic efficacy while minimizing off-target effects. Moreover, these NPs can modulate biological processes relevant to cancer progression, such as angiogenesis and immune response. This review article provides a concise overview of the pathophysiology of breast cancer and the benefits of polysaccharides in drug delivery. Additionally, it emphasizes the significance of several polysaccharide-based NPs in breast cancer therapy, followed by a detailed discussion on the role of various polysaccharide-based NPs in breast cancer treatment.

## 1. Introduction

Breast cancer remains a considerable global health problem, with significant morbidity and mortality rates worldwide. The incidence of breast cancer continues to rise, making it the most commonly diagnosed cancer among women globally [1]. Globally, breast cancer is accountable for a considerable proportion of cancerous tumor cases and cancer-related mortalities among women. According to the latest prevalence statistics from the World Health Organization (WHO), breast cancer is the highest causality of cancer incidence and the second leading rationale of cancer mortality among females worldwide. In 2020, over 2.2 million new cases of breast cancer were diagnosed worldwide, resulting in an estimated 685,000 deaths [2]. In India, breast cancer cases shown a significant public health challenge, with increasing incidence rates observed in recent years [3,4]. Several factors contribute to the high prevalence rate of breast cancer in India, including changing lifestyle patterns, delayed childbearing, reduced breastfeeding duration, and increasing urbanization [5,6].

Many conventional treatments for breast cancer, i.e., chemotherapy, immunotherapy, and radiation therapy, can yield consequential adverse events, including fatigue, hair loss, nausea, and increased risk of infections or low immunogenicity. Additionally, tumor cells may acquire resistance to conventional therapies over time, resulting in treatment failure and disease recurrence [7]. This resistance can emerge due to congenital mutations, tumor heterogeneity, or adaptive transformations within the tumor microenvironment, making it challenging to achieve long-term remission [8]. Despite aggressive treatment regimens, conventional therapies may not completely eradicate the cancer cells, especially in cases of chronic or metastatic disease [7,8]. Therefore, the residual cancer cells can lead to disease relapse and progression, highlighting the need for more effective treatment strategies [9]. In some cases, conventional treatments may be saturated, further expose the patients to unnecessary risks and burdens without significant clinical benefit. This highlights the importance of personalized treatment approaches tailored to individual patient characteristics and tumor biology. Addressing these challenges requires a multifaceted approach, including the development of novel treatment modalities, personalized medicine approaches, supportive care interventions, and efforts to improve access to care and reduce treatment-related inconsistency [10,11,12]. By overcoming these challenges, we can improve the effectiveness, safety, and quality of breast cancer treatment, ultimately enhancing patient outcomes and survivorship. Therefore, breast cancer remains a substantial global health challenge, necessitating the development of innovative treatment strategies.

Nanotechnology has emerged as a promising approach, offering targeted drug delivery and improved therapeutic efficacy, while simultaneously reducing systemic toxicity [13]. In this context, polysaccharide-based nanoparticles (NPs) developed using natural sources have attracted significant attention due to their excellent biocompatibility, structural versatility, and easily tunable properties. Unlike many synthetic polymer-based NPs, which may exhibit long-term toxicity, and lipid-based NPs, which often reported with limited stability, polysaccharide-based NPs offer a safer and more stable alternative, making them highly promising candidates for breast cancer therapy. Polysaccharide-based NPs, i.e., starch, alginate, hyaluronic acid (HA), dextran, cellulose, chitosan (CS), etc., possess inherent biocompatibility and can be functionalized for targeted drug delivery [14,15]. The mucoadhesive properties of polysaccharide-based NPs enable effective uptake at the tumor site, while their biodegradability ensures minimal toxicity [16,17,18]. Polysaccharide-based NPs hold significant promise for breast cancer aid due to their promising physicochemical characteristics and versatile mechanical properties. By functionalizing their surfaces with ligands, polysaccharide-based NPs can specifically target receptors overexpressed on breast cancer cells, improving therapeutic efficacy while minimizing the off-target effects [19,20]. Polysaccharide NPs can be functionalized with proteins or ligands that selectively affix to receptors overexpressed on breast cancer cells, facilitating their uptake and accumulation within the tumor microenvironment [19]. In addition, these NPs may exert additional therapeutic effects through modulation of biological processes relevant to cancer progression, i.e., angiogenesis, apoptosis, and immune response [19,20]. Several polysaccharide NPs may inhibit angiogenesis of cancerous cells via targeting vascular endothelial growth factor (VEGF) receptors [21] or enhance immune infiltration into the tumor microenvironment [22], leading to improved antitumor or anticancer immune responses [22]; therefore, can be combined with other treatment modalities, i.e., radiation therapy, immunotherapy, or chemotherapy, to achieve synergistic therapeutic effects [23]. Thus, polysaccharide NPs can improve cancer therapy efficacy and reduce recurrence risk by enhancing drug delivery and overcoming resistance mechanisms.

To the best of our knowledge, only a few review articles have comprehensively addressed the role of polysaccharide-based NPs in breast cancer therapy [24]. A 2024 review primarily focused on the use of polysaccharide-based NPs for antisense oligonucleotide delivery [25]. Therefore, this review article aims to provide an updated overview of recent advances in various polysaccharide-based NPs designed for the delivery of diverse therapeutic agents, including chemotherapeutic, targeted, phytochemical, and immunotherapeutic agents. This comprehensive manuscript further examines both in vitro and in vivo findings that highlight their efficacy and translational potential. Collectively, polysaccharide-based NPs emerge as promising platforms for targeted breast cancer therapy, offering new avenues to enhance breast cancer treatment outcomes.

## 2. Pathophysiology of Breast Cancer

Breast cancer arises due to mutations in genes that are pivotal for controlling cell division, cell cycle, programmed cell death (apoptosis), and frequently implicated genes include TP53, PIK3CA, MYC, PTEN, CCND1, ERBB2, FGFR1, and GATA3 [26]. Beyond genetic alterations, epigenetic changes, such as DNA methylation and modifications to histone proteins, significantly contribute to the initiation and development of breast cancer [27,28]. These epigenetic changes are reversible, making them highly appealing for therapeutic exploration. The role of estrogen in breast cancer development is substantial, as it strongly influences breast tissue during hormonal transitions such as adolescence, pregnancy, and the menstrual cycle. Estrogen enhances cell growth in breast tissue, thereby increasing the probability of genetic mutations that can initiate cancer. Furthermore, estrogen is particularly associated with driving the growth of cancers that rely on estrogen signaling, especially ER-positive breast cancer [29]. A crucial molecular player in breast cancer is human epidermal growth factor receptor-2 (HER2). HER2, a member of the tyrosine kinase receptor family, activates several intracellular pathways upon stimulation. These include the RAS pathway and PI3K-AKT-MAPK pathway, which collectively promote cell proliferation, survival, and the spread of cancer to other parts of the body [30]. The immune system also interacts with tumor microenvironment in complex ways. Initially, the immune system recognizes and attacks tumor cells by targeting neoantigens produced by mutations [31]. However, chronic exposure to these antigens leads to the activation of immune checkpoint molecules such as CTLA-4 and PD-L1, which suppress immune responses. This suppression allows immunosuppressive cells to accumulate at the tumor site, fostering an environment for tumor survival [32,33,34].

## 3. Importance of Nanotechnology in Breast Cancer Treatment

The growing field of nanotechnology presents a promising outlook for diagnosing, imaging, and treating cancer [35,36]. Breast cancer, one of the significant or prevalent malignancies impacting women’s health worldwide, has particularly benefited from the advancements in nanotechnology. Nanotechnology has revolutionized the prior or premature detection and diagnosis of breast cancer microenvironment through the development of highly sensitive and specific nanoscale imaging agents [37,38,39,40]. NPs functionalized with targeting ligands, i.e., antibodies or peptides, can specifically bind to cancer biomarkers, enabling precise detection using various imaging techniques, i.e., positron emission tomography (PET), magnetic resonance imaging (MRI), computed tomography (CT), etc. [40]. Additionally, nanosensors capable of detecting cancer-related biomolecules in biological fluids offer non-invasive diagnostic approaches with high accuracy and sensitivity [41]. Theranostic NPs enable progressive imaging and treatment of breast cancer via integrating diagnostic and therapeutic functionalities or payloads in a single platform. By incorporating imaging agents (e.g., fluorescent dyes, contrast agents) and therapeutic payloads (e.g., chemotherapeutic drugs, small interfering RNA), theranostic nanomaterials facilitate personalized medicine by providing real-time monitoring of treatment response and disease progression [42,43].

One of the significant challenges in cancer treatment is achieving targeted drug delivery to tumor sites while minimizing systemic toxicity. NPs serve as versatile carriers for anticancer therapeutics, allowing for targeted delivery to breast tumors [39,40]. Surface modification of NPs with ligands specific to overexpressed receptors on cancer cells enhances their accumulation at the tumor site through active targeting mechanisms [38,39,40]. NPs sensitive to stimuli can be specifically designed to release the therapeutics due to the changes in pH, temperature, or enzymatic activity. This “smart” stimuli-drug delivery technique results in a higher drug concentration at the tumor site, effectively reducing off-target effects and enhancing the therapeutic effectiveness of the anticancer agents [44,45]. Using a NP-based technique may avoid problems associated with macromolecules, i.e., lack of permeation, cell toxicity, lack of specificity, and high doses [38]. On the other hand, concerns associated with multidrug resistance (MDR) and P-glycoprotein (P-gp) efflux may potentially undergo certain modifications. By utilizing nanotherapeutic strategies, medical professionals can target cancer cells with unprecedented precision and accuracy, resulting in improved patient outcomes and a brighter outlook for those affected by this devastating disease. These include photothermal therapy (PTT) and photodynamic therapy (PDT), which utilize light-absorbing NPs to generate localized hyperthermia or reactive oxygen species (ROS), respectively, for tumor ablation [13]. Nanovaccines and immunomodulatory NPs are also promising immunotherapeutic solutions for breast cancer, further encouraging the immune system to identify and combat cancerous cells, resulting in precise and effective treatment options [46].

## 4. Polysaccharides—A Multifunctional Polymer for Innovative Nanoparticle Design

Polysaccharides constitute a vital class of hydrophilic polymers originating from natural sources, widely recognized for their intrinsic biocompatibility and functional versatility. Their extensive integration into water-based polymer systems and nanotechnology arises from their remarkable attributes, including biodegradability, non-toxicity, and compatibility with biological systems [47]. These characteristics are critical for the effective application of NPs, positioning polysaccharides as an optimal category of materials for NP development. Polysaccharide-based NPs exhibit significant advantages, such as enhanced drug loading capacity, rapid drug release profiles, precise targeting capabilities, high stability, and minimal toxicity under physiological conditions. These properties not only enhance their suitability for drug delivery but also underscore their relevance in environmentally sustainable and advanced technological innovations [48]. Furthermore, beyond their inherent biodegradability and biocompatibility, polysaccharides have garnered substantial interest due to their natural abundance, ease of processing, and availability from renewable resources, demonstrating their role as a sustainable choice for diverse biomedical and tissue engineering applications [49,50]. Furthermore, polysaccharides undergo chemical functionalization primarily through the activation of their inherent carboxyl and hydroxyl groups, which are distributed along their structural backbone. By leveraging the reactivity of these functional groups, it becomes possible to materialize the polysaccharide derivatives with precisely controlled characteristics, such as reduced hydrophobicity or improved solubility [51]. This targeted modification significantly enhances the versatility and applicability of polysaccharides, particularly in the development of NP-based drug delivery systems, including those specifically designed for cancer therapy [52,53,54].

## 5. Polysaccharide-Based Nanoparticles for Breast Cancer Therapy

### 5.1. Chitosan

CS, also known as chitin, is a polymeric structure that consists of *N*-acetyl-D-glucosamine and D-glucosamine units of the polysaccharide category [55]. Polyionic polysaccharides include several desirable characteristics, i.e., biodegradability, biocompatibility, enhanced cell membrane permeability, and cellular absorption, which further contribute to various biomedical applications. CSNPs possess mucoadhesive properties that enable them to encapsulate or coat therapeutic substances on their surface, thereby controlling the therapeutic or drug delivery to the targeted site. The CS nanocarrier releases the drug when it becomes protonated at a pKa value of 6.4. Cancer cells exhibit a lower pH of 5, which causes the drug to adhere to the negatively charged mucosal surface through electrostatic contact. This results in an improved binding of the drug to the target cells, making it more effective for cancer treatment [56,57]. The anti-metastatic effectiveness of the CSNPs can be attributed to their capability to permeate epithelial cells, resulting in augmented drug permeability and subsequent disruption of tight junctions at the molecular level [58,59,60,61].

The premature detection and instantaneous treatment of breast cancer are strongly recommended using advancement of NPs for therapies and diagnosis of breast cancer. On this note, Ekinci et al. developed methotrexate (MTX) and Tc [99mTc] radionuclide-loaded CSNPs via an ionotropic gelation method for characterizing the potential efficacy and biodistribution of the NPs in breast cancer treatment. The investigation demonstrated that the MTX-CSNPs exhibited a mean hydrodynamic diameter ranging from 148 to 157 nm, a significant polydispersity index (PDI) value (from 0.415 to 0.451), zeta potential (from 15.7 to 18.7 mV), with an MTX % of encapsulation efficiency (%EE) ranging from 64% to 70%. The Tc [99mTc]-MTX-CSNPs exhibited a high labeling capacity (99%) and demonstrated stability up to 6 h at room temperature. The optimized NP exhibited a significant absorption into the 4T1 cancer cell line compared to R/H-[99mTc] Tc. The in vivo result obtained using BALB/c mice exhibited that the cancer cells shown a more significant absorption of Tc [99mTc]-MTX-CSNPs compared to Tc [99mTc] alone. Thus, this study further furnished additional evidence advocating the diagnostic and therapeutic potential treatment of breast cancer using CS-based NPs [62].

Several drug delivery scientists have expressed significant attraction to nanotherapeutic delivery systems based on essential oils conjugated with polysaccharide-based polymeric systems, primarily due to their synergistic anti-inflammatory and pro-angiogenic properties for breast cancer. To harness the challenges of conventional therapeutics or delivery systems, Xu et al. assessed the anticancer potential of essential oil derived from *Cinnamomum cassia* (CEO) encapsulated in CSNPs (CS-CEO). The study determined that the spherical and uniform CS-CEO NPs exhibited a smaller particle size (215.40 ± 3.90 nm), zeta potential (51.70 ± 1.90 mV), higher %EE (83.37 ± 0.4%), drug loading capacity (26.42 ± 0.65%), and demonstrated high stability at 4 °C temperature. The in vitro anticancer efficacy of CS-CEO NPs was validated by the higher expression of Caspase-3 and AIF protein at a concentration of 52 μg/mL. The researchers additionally documented the suppression of tumor cell proliferation, apoptosis, and other related processes in 4T1 breast cancer cells. Therefore, this finding furnishes evidence for the CEO’s physical stability and effective encapsulation within CSNPs, which can be demonstrated as a significant phytomedicine for treating breast cancer, as documented in traditional medicine [63].

The transportation of restoratives or drugs to cancer cells possess significant challenges, mainly due to the constraints related to physicochemical attributes (i.e., low %EE, poor stability, rapid degradation, and bio-distribution) of the delivery system. In order to address these constraints, Rajaei et al. developed 5-fluorouracil (5-FU) loaded CS/agarose/graphene oxide (CS/AG/GO)-based hydrogel system via a double emulsification technique (i.e., w/o/w) for the breast cancer treatment. The resultant nanocomposite exhibited several desirable properties after double emulsification, including an average nanosized particle size of 197 nm, PDI value (i.e., 0.34), high zeta potential (+23.5 mV), %EE (97%), and drug loading capacity (57%). The investigators additionally documented the continuous release of 5-FU at a pH of 5.4 over 48 h and demonstrated the efficient cytotoxicity of CS/AG/GO/5-FU against the MCF-7 cell line, resulting in a 23% reduction in cell viability. These findings further concluded that CS/AG/GO-based hydrogel acted as a pH-sensitive drug delivery system and further enhanced the in vitro delivery efficiency of 5-FU against breast cancer [64].

In the field of cancer treatment and diagnosis, CSNPs have received significant consideration due to their diverse biological and physicochemical features, i.e., glycol-based CSNPs exhibited low immunogenicity, minimal or no toxicity in living organisms, and substantial biodegradability and biocompatibility. However, there are no significant investigations available on the dose-dependent study of these NPs. In this context, Chang et al. synthesized glycol CS conjugated with 5β-cholanic acid, which exhibited NPs with uniform size distributions (i.e., 265.36 to 288.3 nm) under aqueous conditions. In a dose and time-dependent rationale, the researchers observed a notable increase in cellular uptake in the 4T1 and H9C2 cardiomyocyte cell lines compared to macrophages and fibroblast cell lines. This absorption resulted in severe cell necrosis under conditions that were clinically relevant and highly concentrated. The in vivo intravenous administration of glycol-based CSNPs (90 mg/kg) to healthy mice exhibited significant biodistribution or accumulation in the major organs, i.e., heart, after 6 h and remained located for a sustained period of 72 h. This study found that the repeated administration of high doses of glycol-based CSNPs can lead to severe damage to the heart, including inflammation, tissue necrosis, fibrotic changes, and impaired organ function. The findings demonstrate that CSNPs should be utilized with caution in therapeutic environments, and further, the toxicological framework demonstrated the safe integration of CSNPs into breast cancer treatments [65].

Several investigations reported that HA exhibited a high affinity for cell surface receptors, i.e., CD44 has emerged as a prevalent ligand to coat CSNPs to target breast cancer via facilitating the access of NPs into cells and enhancing the concentration of medicine within cancer cells via endocytosis [66,67]. Therefore, Meylina et al. strive to develop HA coated CSNPs to deliver Alpha Mangostin (AM) (AM-CS/HA) specifically to the MCF-7 cell line. The AM-CS/HA exhibited a spherical shape, average diameter (i.e., 304 nm), PDI value (i.e., 0.3), positive zeta potential (i.e., +24.43 mV), %EE (90%), and drug loading (8.5%). The AM-CS/HA exhibited a continuous release of AM at an acidic pH (5.5), surpassing the physiological pH (7.4) and beyond the release observed in an acidic pH of 5.5. The cytotoxicity of AM-CS/HA on MCF-7 cells demonstrated significantly higher (at 4.37 g/mL) compared to AM NPs without HA coating (AM-CS; 4.48 g/mL) and non-coated AM (5.27 g/mL). The AM-CS/HA system enhances the ability of AM to kill cancer cells and could be useful in treating breast cancer. The study suggested that the targeted delivery of AM via NPs has the potential to be a practical therapeutic approach for breast cancer treatment. However, additional research is necessary to assess the bioavailability, toxicity, and anticancer efficacy of AM-loaded CSNPs coated with HA using in vivo introspections [68].

In a recent year, there are multiple investigations reported on CS-decorated or based nanoparticulate systems, i.e., 4-carboxy phenyl boronic acid (4-CPBA)/Palbociclib (PALB)/CS lipid NPs [69], CS/Piperine (PIP)/metal–organic framework (MOF) [PIP@MIL-100(Fe)] [70], CS/Bismuth oxide NPs/Curcumin (CUR)/5-aminolevulinic acid nanocomposite [71], Folate-functionalized carboxymethyl CS (CMCS)/calcium phosphate (CF/CaP) NPs [72], CS/ZnO NPs/Cisplatin nanoconjugates [73], Mesoporous silica/CS/Polyethylene glycol-MUC1 aptamer [74], 5-FU/CS-bimetallic (Au/Pd) NPs [75], etc., have shown substantial potentiality due to favorable characteristics (as mentioned earlier), and binding affinity to the receptors in breast cancer therapy.

### 5.2. Alginate

Alginate, a copolymeric structure, is derived from the combination of L-guluronic acid (G) and D-mannuronic acid (M), further created MG blocks. Alginate is commonly obtained from marine algae and other plant species, including *Laminaria digitata*, *Laminaria saccharina*, and *Macrocystis pyrifera*, and retains a gelling capability that confers both mechanical strength and flexibility, enabling it to withstand moisture present at the drug delivery application site effectively [76]. Alginate NPs provide enhanced drug loading and target-based delivery capabilities, positively reasonable for application in cancer treatment. In the past year, Ahamady et al. materialized an optimized system that incorporated capsaicin-loaded alginate NPs within polycaprolactone-CS nanofiber mats. The alginate NPs further demonstrated significant capsaicin loading, particle size (19.42 nm), and higher %EE (98.7%). The study reported an electrospinning technique to fabricate polycaprolactone-CS nanofibers with varying ratios (100:0, 80:20, and 60:40), and further in vitro characterization on MCF-7 human breast cells provided additional evidence supporting the efficacy of the NPs in inhibiting the proliferation of cancer cells compared to human fibroblast cells. Therefore, this preliminary investigation further provides optimism for the regulation of capsaicin release for more than 120 to 500 h using alginate NPs, which could prove to be efficacious if further examined in vivo and clinical settings in the context of breast cancer treatment [77].

Exemestane (EXE) is a popular and conventional oral medication utilized to treat breast cancer. However, its side effects and reduced bioavailability are causes for concern. As a practicable solution, Jayapal et al. suggested combining alginate NPs with EXE via gelation technique, which can potentially lessen side effects, regulate drug release, and enhance the interaction between the drug and its target receptors. The research findings yielded a particle size of 197 nm, a higher Zeta potential (−18.3 mV), an approximate %EE (i.e., 98%), and a drug release duration of up to 7 h. The study demonstrated that the cytotoxicity analysis on Dalton’s lymphoma ascites cells (DLA) indicated that the alginate NPs loaded with different concentrations (i.e., 10 to 200 µg/mL) of EXE exhibited the highest inhibitory effect on the cancerous cells. Therefore, it has been demonstrated that alginate NPs serve as effective nanocarriers for loading EXE anticancer medication, resulting in prolonged release in alkaline conditions (pH 7.4). Thus, this in vitro study further proves the efficacy and improved target-receptor binding capacity of alginate NPs-loaded therapeutics for breast cancer treatment [78].

Mastectomy is the most effective treatment for breast cancer, as demonstrated clinically. However, it comes with significant challenges, including a high rate of tumor recurrence and the adverse effects of chemotherapy. Hence, it is imperative to develop a therapeutic approach that may efficiently facilitate the process of postoperative wound healing and impede the recurrence of local tumors. Therefore, Su et al. strived to construct a local drug delivery platform called CCNACA, which utilizes carbon dots-CUR nano-drug release (CCNPs) as a 3D printing scaffold. The platform was constructed utilizing CCNPs, sodium alginate, nanoclays, and caffeic acid grafted CS as raw materials. The primary objective of this platform was to enable the visualization of the % cumulative release rate. The in vitro drug release study conducted over 14 days showed that the CCNACA scaffolds exhibited a tumor inhibition rate of 73.77% ± 1.68% on breast cancer cells (MCF-7). Furthermore, the authors demonstrated that introducing CCNACA scaffolds into surgical incisions can effectively suppress remaining cancer cells after surgery, diminish inflammation, stimulate the growth of new blood vessels, and restore damaged tissues resulting from surgical procedures. Thus, the investigation further proposed a local therapeutic delivery system that has significant promise in the realms of wound healing and tumor recurrence prevention after breast cancer surgery [79].

The current research focuses on advancing inorganic NPs-based biomedicine for anticancer therapy due to their cellular absorption significantly influencing the efficacy of inorganic NPs as anticancer agent. In this scenario, the cellular uptake of inorganic NPs is contingent upon various physical and chemical characteristics, i.e., dimensions, morphology, surface modification, passive diffusion, and endocytosis of the NPs. Recently, Karthikeyan et al. investigated inorganic SnO_2_ (tin dioxide) and sodium alginate (SA) into SnO_2_ (SASnO_2_) using an one-pot green technique, which further possesses superior antibacterial, antioxidant, and anticancer characteristics compared to SnO_2_ NPs. The study reported that the in vitro MCF-7 cells exhibited greater cellular uptake of SASnO_2_ NPs (19 nm) than SnO_2_ NPs (38 nm). Therefore, this contemporary investigation further concluded that the increased surface area of these SASnO_2_ NPs facilitates more significant interaction with biological membranes and internalization by cancer cells, leading to improved efficacy in breast cancer therapy [80].

The utilization of magnetic drug targeting has surfaced as a viable approach for the efficient delivery of phytochemicals in the context of breast cancer. Therefore, Bulatao et al. investigated the cytotoxicity of lutein (LUT) loaded CS/alginate iron oxide NPs (LUT-CS/Alg-Fe_3_O_4_-NPs) against breast cancer cells, further optimized via Box–Behnken design considering various dependent variables. The study reported that the optimized LUT-CS/Alg-Fe_3_O_4_-NPs demonstrated biocompatibility and exhibited a notable increase in cytotoxicity towards MCF-7 cells when exposed to a permanent magnet. The produced NPs were confirmed to be superparamagnetic based on their remanent magnetization and minimal magnetic coercivity. Furthermore, the increase in magnetic activity was found to be four times greater than that of free LUT. Therefore, these findings imply that LUT-CS/Alg-Fe_3_O_4_-NPs possessed significant effectiveness for magnetically targeted delivery systems for effective therapy of breast cancer [81].

The three-dimensional (3D) hydrogel structure is fascinating in terms of various physicochemical and mechanical properties. In this context, Ziaei et al. modulated a pH-sensitive in situ structure using oxidized alginate and gelatin-containing doxorubicin (DOX) loaded CS/gold NPs (CS/AuNPs)-based nanogels. The study resulted in satisfactory size distribution (209 nm), positive zeta potential (+19 mV), and higher %EE (72.6%) of DOX. The viscoelastic properties of hydrogels exceed the viscous properties of all hydrogels across the specified frequency range. Further analysis of the mechanical and textural properties demonstrated that β-GP and CS/AuNPs nanogel displayed exceptional mechanical properties. The results of the MTT analysis indicate that the hydrogels exhibited cytocompatibility with MCF-7 cells. The Live/Dead cell assay further revealed that the grown cells on hydrogels without DOX exhibited a high degree of viability when exposed to CS/AuNPs nanogels. The findings from the study indicated that both the hydrogel-encapsulated medication and unbound DOX at equivalent concentrations were able to induce significant apoptosis in the MCF-7 cell line. This suggests that the hydrogels developed in the study have a promising potential for localized breast cancer therapy [82].

In past few years, several investigations reported, i.e., MTX/alginate NPs [83], Iron oxide/sodium alginate/thymoquinone nanocomposites [84], Chitosan/alginate-based platelet/DOX nanoconjugates [85], Capsaicin/alginate NPs/polycaprolactone-chitosan nanofibers [77], Cisplatin/DOX/niosomal alginate nanocarrier [86], Alginate-coated gold nanorods (AuNRs) [87], Gelatin/alginate/DOX-loaded niosomal (Nio-DOX@GT-AL) nanocomposite [88], Alginate/chitosan/Molybdenum nanocomposite [89], Turmeric/alginate NPs [90], Metformin/CUR/chitosan/alginate NPs [91], etc., have shown substantial efficacy against breast cancer cells. However, the bioavailability and pharmacokinetic parameters of multi-drug treatment in breast cancer have not been explored in vivo models.

### 5.3. Hyaluronic Acid

HA is a biological polysaccharide comprising *N*-acetyl-D-glucosamine and D-glucuronic acid and has been demonstrated to be biocompatible, biodegradable, non-immunogenic, non-toxic, and can target CD44 receptor overexpression in tumor cells. Due to these properties, HA possess potential effectiveness for biomedical and pharmaceutical applications. HA has improved drug retention time and delivery profiles when incorporated into developing nano-sized drug delivery systems (DDSs) [92]. 

In a recent investigation, Omar et al. developed α1-acid glycoprotein (AGP)-conjugated HA (AGP-HA NPs) to modulate the responsiveness of breast cancer towards chemotherapy and inhibit the spread of breast tumors. The outcome of in vitro experiments conducted on MCF-7 cells demonstrated the significant efficacy of AGP-HA NPs in inhibiting of tumor migration by 79% up to 24 h. The study showed that AGP-HA NPs effectively regulated MDA-MB-231 cells and restored their sensitivity to the chemotherapeutic drug, i.e., DOX. Additionally, the results obtained using flow cytometry and confocal spectroscopy revealed that the AGP-HA NPs exhibited an increased capacity for DOX absorption and retention, facilitating its delivery to the cell nucleus within a 4 h of incubation period. Therefore, the investigation concluded that HA-based NPs shown a promising and efficacious therapeutic alternative for enhancing anticancer properties of chemotherapeutic molecules, and may potentially enhance the survival rate if investigated in vivo [93].

Recently, the combination of chemotherapeutic agents has demonstrated greater effectiveness in treating multi-drug resistance cancer (MDRC). Thus, in order to target the MDRC treatment, Thummarati et al. designed dual pH-responsive NPs composed of gemcitabine (GEM) and CUR onto HA using a solvent diffusion method. The conjugates developed based on ester and hydrazone bonds, leading to the materialization of GeChH (random graft) and (GhC)hH (block graft). The study demonstrated that average particle size (221 nm), narrower PDI value (0.01), and release profile of GEM and CUR from the nanoconjugates exhibited faster drug release in the acidic microenvironment, whereas controlled at physiological pH (i.e., 7.4), suggesting pH-dependent behavior. Furthermore, the cytotoxicity study of GhChH NPs exhibited more significant cytotoxicity and a synergistic effect in various cell lines (i.e., Caco-2, HCT116, PANC-1, and A549) compared to GeChH NPs and GEM/CUR suspension. In addition, the % of uptake of the (GhC)hHFITC NPs demonstrated higher values or numbers compared to GeChHFITC NPs in A549 and HCT116 cell lines. By employing an HA blockade, the NP uptake percentage experienced a notable decrease, indicating the pivotal role of CD44 receptor targeting by HA in enhancing the specificity of the cancer cells. Thus, these newly developed NPs can potentially deliver both GEM and CUR in a pH-responsive manner, which could be advantageous for treating breast cancer [94].

In recent times, a substantial body of research has emerged, demonstrating a strong correlation between breast cancer and vitamin D3 (VD3). Nevertheless, there have been limited investigations into the development of VD3-loaded delivery approaches targeting breast cancer. Therefore, Gao et al. characterized the conjugation of VD3 and HA (HA-VD) to effectively target breast cancer by facilitating the administration of DOX. The developed formulation further confirmed the % degree of substitution of 18.6%, whereas, at the molar concentration ratio of 1:2, the formulations possessed 0.0137 mg/mL of critical micelle concentration. The HA-VD micelles loaded with DOX (DOX/HA-VD) shown 6.2% of drug loading, with an %EE of 79.5%, and further demonstrated as viable or stable in blood for 48 h. The % cumulative release of DOX exhibited a reduction following encapsulation compared to the release rate seen in the absence of HA-VD. The decrease in IC50 of the DOX/HA-VD micelle showed a statistically significant difference compared to free DOX and blank micelles. VD3 and DOX had a synergistic effect on BC cells at a cell inhibition rate of 50%. Therefore, the utilization of HA-VD micelles has been shown to significantly enhance the therapeutic efficacy of DOX in targeting breast cancer [95].

In another investigation, Zeng et al. highlighted the synergistic effects of dasatinib (DAS) and olaparib (OLA) on triple-negative breast cancer (TNBC) via pH-sensitive ester bonds to establish a connection between DAS and HA (HA-DAS). Subsequently, the researchers demonstrated that the incorporation of OLA and HA-DAS served as the carrier for synthesizing nanomicelles (HDO-NPs). The physicochemical characterization study indicated that HDO-NPs exhibited a spherical shape, monodispersed particle size, excellent stability, etc. Furthermore, cellular investigations demonstrated that the HDO-NPs were efficiently absorbed by attaching to the CD44 protein overexpression of MDA-MB-231 cells, leading to a higher drug concentration within the cells. The results of the in vivo investigation exhibited that the HDO-NPs exhibit efficient targeting of tumor tissues and remarkable therapeutic efficacy against tumors. Additionally, these NPs greatly extend the in vivo circulation period of pharmaceuticals and successfully enhance drug bioavailability, therefore exhibiting a promising potential for utilization in the therapeutic management of TNBC [96].

Several substantial investigations on HA-based NPs in laboratory settings, i.e., HA/poly(methacrylic acid)/mesoporous silica nanocomposite [97], Chemodynamic therapy (CDT) agent (HA-Fc-Mal)/HA NPs [98], HA/copper sulfide NPs/diethyldithiocarbamate (DDTC)/losartan nanocomposite [99], HA/manganese dioxide (HMnO2)/enzalutamide/MK-8776 nanoconjugates [100], Dopamine-reduced GO nanomaterials/HA/DOX NPs [101], HA modified chitosan/chebulinic acid NPs [102], HA-coated berberine (BBR)/indocyanine NPs [103], etc., have been documented that the HA-based NPs loaded several therapeutic compounds possess improved anticancer potentiality in breast cancer treatment.

### 5.4. Dextran

Several NPs based on dextran exhibit potential carriers or drug delivery systems for treating breast cancer owing to various favorable attributes, including biocompatibility, biodegradability, and a high surface area-to-volume ratio. These characteristics enable the improved loading of therapeutic agents, i.e., chemotherapeutic drugs, peptides, or nucleic acids. Dextran NPs can permeate through blood vessels and inadequate lymphatic drainage typical of solid tumors. This permits to accumulate in the tumor tissue through the improved permeability and retention capability consequence. Dextran NPs provide a flexible platform for surface modification, facilitating the integration of imaging agents for diagnostic applications or therapeutic agents for combination therapy. Furthermore, it is worth noting that dextran NPs often elicit limited immunological responses, hence decreasing the probability of unfavorable reactions or immune-mediated elimination following their administration.

The emergence of TNBC has revealed numerous therapeutic targets, indicating that a single target for the treatment may be unsuccessful, and has further emphasized the necessity to innovate substantial technologies for combination therapy. Paclitaxel (PTX), widely recognized as a groundbreaking development in the field of chemotherapy, encounters various challenges stemming from its limited solubility and permeability. The significance of interferons in cancer has been elucidated by ongoing scientific advancements. Therefore, Bakrania et al. developed DEAE-Dextran-loaded PTX NPs to induce interferon in TNBC treatment. The study exhibited significant synergistic cytotoxicity across different cell lines and further suppressed ROS by inducing β-interferon, countering ROS activation by PTX. The nanoformulation was additionally linked to FITC for internalization investigations. These tests revealed that the highest level of cellular uptake occurred 60 min after treatment with FITC, which illuminated the nuclear membrane. The in vivo xenograft model was developed to examine the mechanistic understanding of nuclear-targeted nanoformulation and demonstrated a synergistic release of β-interferon at the targeted site. Furthermore, the combinatorial nanoformulation induced various mechanisms by inhibiting VEGF and NOTCH1 and by overexpressing both β and β-interferon. Thus, the combination therapy shows potential as a versatile nanomaterial for delivering drugs into the nucleus of TNBC [104].

Dextran NPs provide a flexible platform for surface modification, facilitating the integration of imaging agents for diagnostic applications or therapeutic agents for combination therapy. To utilize this advantage, Wang et al. developed carboxymethyl dextran (CMD) loaded PTX/docosahexaenoic acid (DHA) for the conjugation, which resulted in CMD-DHA-PTX-(conjugate S, and conjugate L). The study resulted in conjugates (i.e., Gly Gly and Lys-Gly Gly) exhibiting elevated PTX loading capacity, improved water solubility, and the capacity for self-assembly into NPs (88.7 nm to 94.7 nm), further exhibiting continuous release of PTX in blood and tumor cells. In addition, conjugate S demonstrated enhanced pharmacokinetic characteristics and more excellent tumor site distribution compared to the original PTX, conjugate L, and abraxane. The two conjugates demonstrated superior anti-tumor effectiveness compared to the leading PTX formulation and abraxane in MCF-7-induced nude mice. The administration of conjugate S therapy resulted in the complete eradication of xenograft tumors without inducing any reduction in body weight. This study suggested that the materialization of dextran-based dual-chemotherapeutic conjugates shows excellent potential and innovation in delivering anticancer molecules to various types of tumors [105].

Similarly, Li et al. investigated the potential of cisplatin-loaded LHRH-modified dextran NPs (Dex-SA-CDDP-LHRH) to effectively suppress primary tumor development and metastasis. The study demonstrated that Dex-SA-CDDP-LHRH NPs effectively controlled the targeting ability of LHRH and selectively affixed to the LHRH receptors. The Dex-SA-CDDP-LHRH NPs exhibited superior cellular absorption and heightened cytotoxicity compared to the non-targeted Dex-SA-CDDP NPs. In addition, using non-targeted and targeted NPs led to a significant decrease in the overall toxicity of CDDP and an increase in the tolerated dose of CDDP (4 to 30 mg·kg^−1^). The treatment of Dex-SA-CDDP-LHRH led to a substantial augmentation in the accumulation of CDDP within both the primary tumor and the organs harboring metastases, resulting in a significant decrease in the nephrotoxic impacts of CDDP. Therefore, the CDDP/LHRH-decorated NPs exhibited a notable improvement in anticancer and anti-metastasis effectiveness compared to the non-targeted NPs, as evidenced by dose-dependent therapeutic effects. The findings of this study indicate that Dex-SA-CDDP-LHRH NPs exhibit significant promise in targeted chemotherapy for metastatic breast cancer [106].

The presence of drug resistance in cancer treatment significantly hampers the efficacy of chemotherapeutic drugs, resulting in unfavorable clinical results. The majority of chemotherapeutic agents can stimulate autophagy, enhancing tumor resistance to these medicines and diminishing the efficacy of drug transport to tumor cells. In another investigation, Li et al. developed a nanocomposite composed of carboxymethyl β-dextran (CMD)/protamine sulfate (PS) to facilitate the transportation of docetaxel (DTX), chloroquine (CQ), and Atg5 siRNA within cancerous cells. The study demonstrated that the electrostatic interaction drives the CQ + DTX + Atg5 siRNA NPs, further resulting in the encapsulation of CQ and Atg5 siRNA in NPs, which can augment the responsiveness of tumor cells to DTX. The NPs demonstrated significant therapeutic efficacy in TNBC and excellent biosafety. Hence, this novel multifunctional nanoplatform was developed utilizing CMD and PS, which effectively boosts the sensitivity of chemotherapeutic drugs by inhibiting autophagy. This approach presents promising prospects for addressing the issue of conventional drug resistance and improving therapeutic efficacy in TNBC [107].

Several investigations further reported, i.e., Fe_3_O_4_ NPs/dextran (Fe_3_O_4_@Dex)/glucosamine [108], CMD/thienopyridine derivative [109], Streptavidin (SA)-functionalized CMD/melamine NPs (MNPs) [110], Dextran/iron oxide NPs (SDIO) [111], SPIONs/CMD/triptorelin (SPION@CMD@triptorelin) nanoconjugate [112], CUR/poly (lactic-co-glycolic) acid/chitosan/dextran/PEG nanoemulsion [113], CMD/trimethyl chitosan (TMC) NPs/NIK/STAT3-specific siRNA/BV6 [114], Indomethacin (IND)-grafted dextran/DOX nanomicelles [115], etc., utilization of dextran in conjugate or grafting agent to improve the bioavailability and pharmacokinetics properties of several nanoconjugates for the treatment of breast cancer.

### 5.5. Cellulose

Cellulose, a polysaccharide that occurs naturally in plants, exhibits biocompatibility and high tolerability towards cellulose NPs inside biological systems. Cellulose NPs can decompose naturally, exhibit minimal immune response, and can be functionalized with specific ligands or antibodies that specifically attach to excessively present receptors on breast cancer cells. Cellulose NPs possess physicochemical features, including size, surface chemistry, and shape, which can be readily customized using a range of production and modification methodologies. The adaptability of NPs enables them to be tailored to individual needs for precise medication administration, imaging, or other therapeutic uses in breast cancer treatment.

In a recent year, Karimi et al. investigated gefitinib-loaded cellulose acetate butyrate NPs (Gnb-NPs), further loaded into CS/β-glycerophosphate thermo-responsive hydrogels via the solvent evaporation method for intratumoral delivery in breast cancer-induced animals. The study reported that significant spherical particle size (156.50 ± 2.40 nm), zeta potential (−4.90 ± 0.04 mV), %EE (99.77 ± 0.09%), swelling, gelling time, injectability, and release behavior of the formulated Gnb-NPs-Hydrogel were further evaluated for anticancer activity on the 4T1 cell line. The cytotoxic effect of Gnb-NPs-hydrogel was reported to be more pronounced compared to free Gnb and Gnb-hydrogel. Moreover, administering Gnb-NPs-Hydrogel through intratumor injection demonstrated the highest effectiveness against tumors in living organisms. Thus, the Gnb-NPs-hydrogel demonstrated a significant potential effect as a viable breast cancer therapy option [116].

Generally, the chemotherapeutic agents utilized in the treatment of cancer exhibit limited selectivity towards both normal and cancerous cells, thereby exacerbating the associated cytotoxic effects. The key to resolving this problem lies in efficiently encapsulating anticancer drugs; therefore, Fawal et al. addressed the low solubility and selectivity of disulfiram (DS) via loading into cellulose acetate (CA)/poly (ethylene oxide) (PEO) matrices (DS@CA/PEO) via the electrospinning technique for targeting breast cancer. The study reported that fabrication of DS into the CA/PEO scaffold exhibited superior compatibility compared to the DS-free scaffold when tested on normal human cells (Wi-38). Simultaneously, the DS-free scaffold showed comparable anticancer activity against the Caco-2 and MDA-MB-231 cell lines. The greater affinity of DS@CA/PEO for cancer cells compared to normal cells was linked to the preservation of apoptotic activity and the ability of DS to inhibit aldehyde dehydrogenase. This effectiveness resulted in eliminating stem cells from colon and breast tumors, as demonstrated by flow cytometry. Therefore, this investigation examined the potential of the dual-responsive DS@CA/PEO nanofiber scaffold for specific anticancer properties for breast cancer treatment [117].

Breast cancer treatment regimens have transitioned from single agents to combinatorial approaches, providing combined effectiveness and minimizing adverse effects. An extended platform for tissue-specific co-delivery can be provided by self-assembled nanogels that consist of natural polysaccharides and functional proteins. In a recent investigation, Attalah et al. developed lactoferrin (Lf)/CMC-based nanogels loaded with pemetrexed (PMT)/honokiol (HK). The produced Lf-CMC NGs were utilized to encapsulate an inclusion complex consisting of HK and hydroxypropyl-β-cyclodextrin, resulting in a %EE of 66.67%. The cross-linked Lf-CMC NPs demonstrated an average hydrodynamic diameter (193.4 nm) and a negative surface charge (−34.5 mV). The MDA-MB-231 cells effectively absorbed PMT/HK-loaded Lf-CMC NGs, which further exhibited enhanced cytotoxicity. This was evidenced by a low combination index value and a more extensive dose reduction index compared to the free drugs. A study on a mouse model of Ehrlich ascites tumor (EAT) demonstrated the substantial effectiveness of PMT/HK-Lf-CMC NPs in preventing tumor expansion. This was attributed to the improved caspase-3 expression and decreased VEGF-1 and Ki-67 protein expression levels in the cells [118].

A recent study by Salahuddin et al. investigated the antibacterial and cytotoxic effects of methylene blue (MB) loaded extracted cellulose, explicitly targeting the MCF-7 cells. The microscopic images of cellulose obtained through acid hydrolysis exhibited a filamentous structure with a diameter ranging from 30 to 50 nm and a length spanning or fragmentation from 270 to 1290 nm. The drug release study reported 52% of MB release at pH 7.4 up to 1400 h, whereas the changes in the pH (i.e., 5.4 and 6.7) led to lesser or decreased % of release (45% and 42%, respectively). The cytotoxicity assay of MB/cellulose NPs against MCF-7 exhibited % of cell viability decreased from 52.4% to 6.9% due to an increase in the concentration of MB from 12.5 to 100 mg/mL. The antibacterial efficacy of cellulose-loaded MB was assessed against a wide range of bacteria, demonstrating its efficacy against all tested bacterial species [119].

In addition, the recent years further reported several investigations on cellulose-based NPs, i.e., Lignin/MTX/iron oxide NPs [120], Gefitinib/cellulose acetate butyrate NPs (Gnb-NPs) [116], Cellulose nanocrystals (CNCs)/poly(ε-caprolactone-co-lactide)-b-poly(ethylene glycol) (PCLA) [121], 5-FU/MgO/methyl cellulose NPs [122], Silver NPs/Ethyl cellulose (AgNPs-EC) NPs [123], SF/cellulose acetate/gold-silver NPs (CA/SF/Au-Ag) composite nanofiber [124], Nano cellulose/CUR [125], CUR/polyvinyl alcohol/cellulose nanocrystals (PVA/CNCs) [126], etc., have been extensively investigated in various models for breast cancer therapy.

### 5.6. Starch

Starch-based nanomaterials are currently prominent candidates in the field of biomedical research, offering a broad spectrum of potential applications in the area of targeted drug delivery and inhibition of tumor growth. Starch-based NPs possess extensive adaptability, effectively encapsulating a wide range of therapeutic agents. Furthermore, the utilization of ligand-functionalized NPs in starch-based nano-drug delivery systems holds significant promise for cancer therapy. In this study, Mariadoss et al. developed a novel approach to treat TNBC by synthesizing aptamer-laden starch NPs (Apt-p-CA-AStNPs) loaded with p-coumaric acid (p-CA). The investigation utilized the FT-IR technique to validate the functional groups linked to p-CA and amino starch (AS) in p-CA-AStNPs, followed by gel electrophoresis. The results indicated Apt-p-CA-AStNPs NPs exhibited an average zeta potential (29.2 ± 1.35 mV), particle size (218.97 ± 3.07), PDI value (0.299 ± 0.050), and %EE and loading capacity of 80.30 ± 0.53%, and 10.35 ± 0.85%, respectively. The Apt-p-CA-AStNPs demonstrated a burst release within a 5 h period of the experiment under the pH of 5.4. The Apt-p-CA-AStNPs effectively suppressed the apoptosis-related proteins in the MDA-MB-231 cell line by regulating ROS, mitochondrial membrane potential, and DNA damage [127]. Conjugated bio-nanomaterials, including magnetic cores, have emerged as a promising platform for developing advanced biological composites for breast cancer therapy. To utilize the characteristics of such biomaterials loaded magnetic NPs, He et al. developed dual core–shell NP-loaded Ag NPs functionalized with CS-starch composite. The MTT assay of breast cancer cells treated with Fe_3_O_4_@CS-Starch/Ag nanocomposite showed cytotoxicity at IC50 concentration. In the Fe_3_O_4_@CS-Starch/Ag nanocomposite, the % viability of breast cancer cells exhibited a dose-dependent reduction, further exhibiting different IC50 values of 183, 194, 207, 279, and 252 µg/mL against multiple cell lines (i.e., Hs 578Bst, UACC-3133, Hs 319 T, UACC-732, and MDA-MB-453, respectively) [128].

In general, cancer stem cells (CSCs) exhibited self-regeneration, differentiation, and tumor initiation, which are widely acknowledged as the primary cause of therapy resistance, metastasis, and regression. Concurrently eliminating CSCs and large cancer cells is essential for effective cancer treatment. Thus, Xu et al. utilized hydroxyethyl starch-polycaprolactone NPs (DEPH NPs) co-loaded with Dox/erastin for the elimination of CSCs and cancer cells via the regulation of ROS. This resulted in a highly synergistic impact in a combination of Dox and erastin simultaneously administered by DEPH NPs. Erastin can potentially reduce intracellular glutathione (GSH) levels, which might hinder the movement of intracellular Dox and enhance the production of ROS generated by Dox. The elevated amounts of ROS inhibited the self-renewal of CSCs via downregulation of the hedgehog pathway, facilitated the differentiation of CSCs, and made differentiated cancer cells susceptible to necrosis. In addition, DEPH NPs demonstrated notable efficacy in eradicating cancer cells, mainly CSCs, hence playing a crucial role in inhibiting tumor growth, tumor-initiating potential, and metastasis in TNBC. Therefore, this study proves that combining Dox and erastin is significantly effective in eradicating cancer cells and CSCs, showing potential as a treatment for solid tumors with CSCs [129].

There has been significant interest in utilizing prodrug-based nanomedicines that exhibit superior drug loading capacity and tumor targeting at lower doses for cancer treatment, especially for solid tumors, i.e., TNBC. Nevertheless, the anticancer potential of prodrug nanomedicines is significantly hindered by the complex tumor mechanical microenvironment (TMME), which hinders drug delivery and promotes the proliferation of CSCs. In a recent year, Wang et al. developed carbamate disulfide-bridged DOX dimeric/IR780/hydroxyethyl starch-folic acid conjugates to function for theragnostic purposes to target TNBC and achieve drug release, resulting in high GSH concentration in CSCs. The study reported the increased sensitivity of chemotherapy, mediated by FDINs, which further plays an important role in regulating TMME, depleting extracellular matrix proteins. In addition, FDINs effectively eradicate CSCs by challenging the distinct niche of CSCs and depleting intracellular GSH within CSCs. Consequently, FDINs effectively inhibit the growth of tumors in subcutaneous and orthotopic 4T1-induced tumors. Thus, this investigation proffers new perspectives on the significant development of prodrug-based nanomedicines to establish enhanced therapeutic efficacy against TNBC [130].

Epigenetic medicines, e.g., histone deacetylase inhibitors, frequently encounter diminished effectiveness due to their inadequate solubility in water, leading to restricted bioavailability and a lowered therapeutic index. A biocompatible starch NP-loaded CG-1521 is currently considered for potential preclinical development for breast tumors to address the inadequate therapeutic index. The optimized CG-NPs are further characterized for physicochemical properties, i.e., morphology, zeta potential, loading capacity, and release kinetics. Additionally, their cytotoxic and apoptotic behavior has been assessed in the MCF-7 cell line. This study further demonstrated that the encapsulation of CG-1521 leads to a notable decrease in the rate at which the drug is released while also resulting in a significant cytotoxic capacity of the NPs compared to an equivalent dose of free CG-1521. The application of CG-NPs induced cell cycle and substantial cell death in the MCF-7 cell line via apoptosis, further exhibiting a comparable influence on gene expression as that of a free drug. The results indicated that incorporating CG-1521/starch NPs can potentially enhance the distribution of histone deacetylase inhibitors for breast cancer treatment while maintaining the drug’s effective mechanism of action [131].

Several investigations, i.e., Starch/polydopamine coated core–shell NPs [132], Bionized nanoferrite NPs/anti-HER2 antibody (BH NPs) [133], Tyramine-functionalized starch (Tyr-St)/tannic acid (TA)/phenolated-magnetic NPs (Fe_3_O_4_-PhOH NPs)/Dox [134], Hydroxychloroquine (HCQ)/Fmoc/hydroxyethyl starch (HES)/PTX [135], Polyethylenimine-β-cyclodextrin (roFPC)/Dox/hTERT siRNA [136], etc., have been further developed and investigated at in vitro settings to understand the efficacy in breast cancer therapy.

### 5.7. Chondroitin Sulfate

**Figure 1:**
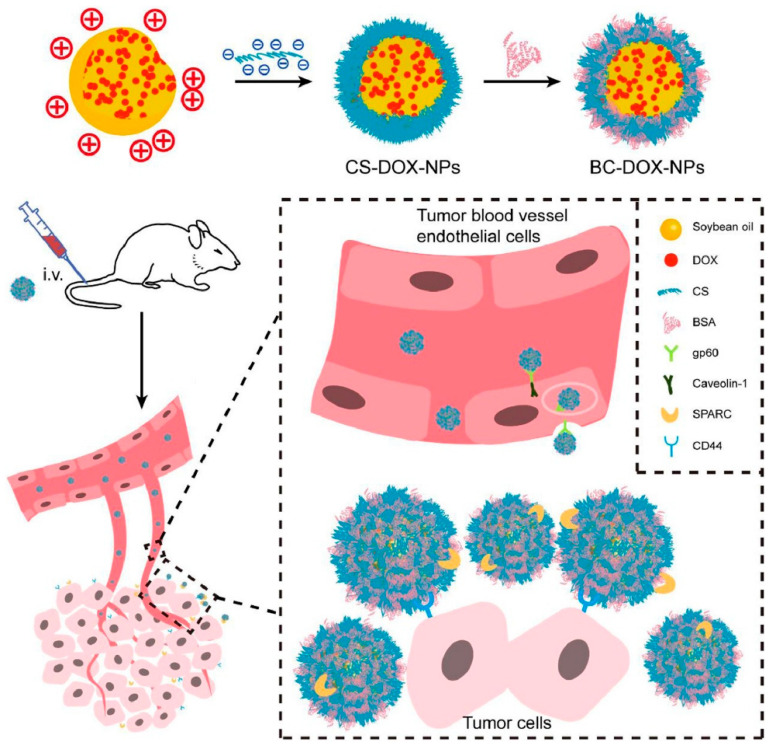
Diagrammatic representation of developed intravenous (i.v) administration of albumin corona NPs-mediated CS/DOX and investigated the efficacy against 4T1 breast cancer cells. BC-DOX-NPs synergized with the gp60, CD44, etc., receptors on tumor cells, resulting in enhanced transcytosis and improved accumulation and uptake inside tumor tissues, reproduced from Ref. [137], licensed under CC BY 4.0.

The utilization of chondroitin sulfate NPs has shown great potential in treating breast cancer, owing to its numerous advantageous features such as precise delivery, encapsulation of anticancer drugs, enhanced drug absorption, and the ability to overcome drug resistance. These NPs are designed to be modified to transport various therapeutic molecules concurrently, hence enabling the occurrence of synergistic effects between medications or combination therapies. Therefore, to utilize these, Tan et al. developed albumin corona NP-mediated chondroitin sulfate-loaded DOX and investigated the efficacy against 4T1 breast cancer cells. The BC-DOX-NPs synergized with the gp60, CD44, and SPARC receptors on tumor cells, resulting in enhanced transcytosis and improved accumulation and uptake inside tumor tissues (Figure 1). This interaction was facilitated by the combined action of BSA and CS. The coexistence of BSA and CS facilitated the effective targeting of CD44 by BC-DOX-NPs, resulting in cytotoxicity in the 4T1 cell line compared to CS-DOX-NPs or free DOX. The administration of BC-DOX-NPs in orthotopic 4T1 tumor-bearing mice resulted in enhanced accumulation of chemotherapeutics at the tumor site compared to CS-DOX-NPs or free DOX. Thus, this investigation further demonstrated the suppression of tumor proliferation and reduced drug deposition in crucial organs in the case of multi-drug treatment or resistance in breast cancer [137].

MDR poses a significant barrier to the efficacy of chemotherapeutics in the treatment of breast cancer. To address the MDR in cancer therapeutics, Shi et al. developed quercetin/chlorin e6/PTX-loaded redox-responsive chondroitin sulfate NPs in the treatment of multidrug-resistant (MDR) breast cancer and lung metastasis. The results of the cell line investigation demonstrated that the presence of NPs led to decreased P-gp expression in MCF-7 and ADR cells. Consequently, this downregulation led to an enhancement in the antitumor effectiveness of PTX against MCF-7 and ADR cells. Furthermore, applying near-infrared (NIR) laser irradiation can stimulate NPs to produce ROS within cells. This, in turn, can result in the disruption of the mitochondrial membrane and facilitate the escape of medicines via lysosomes. This novel nanoplatform demonstrated efficient in vivo MDR suppression and effectiveness in preventing metastasis by enhancing chemo-photodynamic treatment. Thus, this study indicated that the versatile nanoplatform exhibited promising potential for efficient breast cancer treatment [138].

The utilization of polysaccharide-based biomaterials as a delivery system has been recently investigated widely due to their high payload and %EE. In a recent year, Setayash et al. grafted octadecylamine onto chondroitin sulfate (ChoS-ODA1) at different molar ratios (10, 20, and 30)-loaded CUR nanocomposites to treat breast cancer. The study reported that ChoS-ODA3 with 10% CUR was chosen for subsequent testing because it exhibited a higher %EE of 79.56% ± 5.56. The CUR-laden nanogels exhibited an in vitro release profile of over 80% over 70 h. Furthermore, the MTT study of the MCF-7 cell line revealed no significant cytotoxicity towards the nanogel (blank), whereas the CUR-loaded nanogels caused substantial cell death within 24 h. Additionally, the findings of this investigation demonstrated that the utilization of CUR-loaded ChoS nanogels effectively enhanced cellular absorption compared to the administration of free CUR. The nanogels that have been manufactured that incorporate CUR are anticipated to exhibit efficacy in future investigations about cancer therapy [139].

To utilize the receptor-mediated capacity of ChoS-based NPs, Li et al. developed DTX-loaded redox-responsive ChoS to target tumors by attaching to the CD44 receptor, expressed on the surfaces of different tumor cells. Small-molecular DTX is susceptible to redox reactions synthesized via alterations of DTX-loaded cystamine-containing disulfide links (Cys-DTX) nanoconjugates. The study reported that the nanosized DTX delivery is anticipated to possess significant permeability and cytotoxicity of the Cys-DTX NPs, intended to facilitate the targeted transportation of the encapsulated redox-responsive Cys-DTX prodrug. Furthermore, the study observed that the Cys-DTX/ChoS-ss-DTX NPs exhibited a redox-responsive release of DTX via controlled manner. Additionally, these NPs demonstrate enhanced permeability in tissues, heightened cytotoxicity, and reduced adverse events compared to unbound DTX. The findings of this study indicate that the Cys-DTX/ChoS-ss-DTX NPs exhibit promising potential for future application in tumor treatment [140].

Similarly to the objective of the previous investigation, Singhai et al. also developed α-tocopheryl succinate (α-TOS) and ChoS mediated multiwalled carbon-nanotubes (MWCNTs) (α-TOS–ChoS–MWCNTs)-loaded DOX to enable the exact targeting of overexpressed CD44 receptors on cells unique to TNBC. The study reported that α-TOS-ChoS-MWCNTs/Dx composite demonstrated enhanced cellular localization compared to formulations without ChoS, indicating a higher level of selectivity. Notably, the formulation with α-TOS-ChoS-MWCNTs/Dx demonstrated enhanced cellular localization compared to formulations without ChoS, indicating a higher level of selectivity. The Kiton Red 620 assay demonstrated a statistically significant reduction in the proliferation of MDA-MB-231 cells, as indicated by a GI50 value of 0.791 ± 0.015. The investigation on apoptosis utilizing the Annexin V/PI test demonstrated significant apoptosis in MDA-MB-231 cells (53.40 ± 3.32%) when exposed to α-TOS-ChoS-MWCNTs/Dx, as compared to alternative formulations. The results indicated that using ChoS, α-TOS, and Dx combined demonstrated efficacy and safety in treating TNBC [141].

In recent years, several investigations, i.e., Berberine derivative (BD)/chondroitin sulfate (ChoS) [142], PTX/quercetin (QC)/chondroitin sulfate (ChoS)/mesoporous silica NPs (MSNs) [143], HA/chondroitin sulfate (ChoS)/DOX [144], ChoS-deoxycholic acid (ChoS-DOCA) NPs [145], ChoS-deoxycholic acid-polyethylene glycol-maleimide (ChoS-DOCA-PEG-MAL = CDPM) nanostructures [146], Chondroitin sulfate/deoxycholic acid (DOCA) nanocomposite [147], Chondroitin sulfate/polyethyleneimine (BPEI)/BPEI conjugated graphene (BPEI-GO)/Dox [148], Celecoxib/honokiol/chondroitin sulfate/lactoferrin (LF) [149], etc., have been assessed in various models for breast cancer therapy.

### 5.8. Pullulan

Another significant polysaccharide-based NP, i.e., pullan, has shown great potential in treating breast cancer, owing to similar characteristics of other polysaccharides. Therefore, to investigate the potential of pullulan-based NPs, Bonzi et al. developed novel biocomposite-based pullulan NPs loaded with alendronate (ALN) and PTX Pull-(GGPNle-β-PTX) with the specific purpose of specifically targeting bone metastases in breast cancer. The investigation results showed that Pull-(GGPNle-γ-PTX)-(PEG-ALN) exhibited a pronounced attraction towards hydroxyapatite, a material that closely resembles bone tissue. Under pathological conditions, the release of PTX from the bioconjugate was observed rapidly by the cleavage of Cathepsin K. The results of many tests conducted on human MDA-MB-231-BM, murine 4T1, murine K7M2, and human SAOS-2 osteosarcoma cells consistently demonstrated that the bioconjugate exhibited a significant anti-angiogenic activity compared to conjugate ALN. In addition, the nanoconjugate could impede the migration of tumor cells and exhibit significant anti-angiogenic properties. The findings exhibit remarkable promise for targeted therapy of bone neoplasms, including breast cancer, bone metastases, and osteosarcoma [150].

Breast cancer regulates the expression of human epidermal growth factor receptor-2 (HER-2), which is further characterized by its high invasiveness, unfavorable clinical outcomes, and elevated likelihood of recurrence. Therefore, the study investigated by Xu et al. involved trastuzumab functionalized pullulan-DOX (Tz-P-DOX) NPs for targeting the overexpression of epidermal growth factors in breast cancer. The acquired NPs exhibited an average hydrodynamic diameter (6.7 ± 2.0 nm) and PDI of 0.218 ± 0.012. The study findings demonstrated a notable cellular uptake and cytotoxicity between Tz-P-Dox and non-targeted P-Dox when tested in HER-2-positive cell lines, including BT474 and MCF-7 cells. The outcome of this investigation indicated that NPs functionalized with trastuzumab exhibit significant promise as a viable therapeutic option for the treatment of HER-2-overexpressing breast cancer conditions [151].

The primary challenge in integrating chemotherapy and gene therapy lies in developing a viable and biocompatible carrier for the concurrent delivery of therapeutic medication and genes. Therefore, Chen et al. investigated the potential of a novel amphiphilic bifunctional pullulan derivative (PDP) as a nano-carrier for the simultaneous delivery of chemotherapeutics and genes in the context of cancer therapy. The study resulted in favorable blood compatibility and minimal cytotoxicity when exposed to 160.8 nm of particle size and 28 mV of zeta potential of the nanocarrier. The %EE of the drug in PDP micelles was significantly greater (84.05%) compared to the loading capacity of 7.64% for DOX, exhibited a prolonged drug release profile, and demonstrated excellent DNA-binding ability. The utilization of flow cytometry revealed that MCF-7 cells effectively internalize PDP/DOX micelles, resulting in a greater level of cytotoxicity against the MCF-7 cell line compared to free DOX. In addition, it was shown that PDP micelles exhibited adequate transportation of the tumor suppressor gene p53 into MCF-7 cells. Furthermore, the introduction of exogenous p53 protein resulted in the induction of cell death in MCF-7 cells. Furthermore, it was shown that the co-delivery of DOX and gene p53 resulted in increased cytotoxicity, a higher rate of tumor cell apoptosis, and more efficient inhibition of cancer cell migration. The co-administration of DOX and p53 in mice with tumors demonstrated a higher level of effectiveness in combating tumors compared to the individual administration of DOX or p53 alone. These findings indicate that cationic PDP micelles have great potential for efficiently delivering functional genes and chemotherapeutic drugs, enhancing antitumor effectiveness, and reducing systemic toxicity [152].

To minimize the cytotoxicity of chemotherapeutic agents, Asgari et al. fabricated an electrospun composite-based poly (epichlorohydrin) (PCH) grafted GO and further loaded with PTX and Cur, which are subsequently encapsulated into pullulan nanofibers through electrospinning. The compounds exhibited a prolonged release (93 h) from the nanofibers loaded with nanocarriers under pH 7.4. A synergistic cytotoxicity of PTX + Cur was confirmed via MTT assay and optical microscopic study. The nanofiber system, encapsulated with nanocarriers, is an innovative and adjustable drug delivery method designed for localized chemotherapeutic purposes [153].

In addition, very limited investigations have been reported on pullulan-based NPs, i.e., Pullulan/poly(β-amino) ester (PBAE)/MTX [154], Urocanic acid/pullulan/Adriamycin (ADR) [155], Polyethylene sebacate (PES)-Gantrez^®^ AN 119/DOX hydrochloride NPs/pullulan [156], Pullulan (PUL)-Nalpha-Boc-L-histidine (bHis) conjugates/deoxycholic acid (DO) [157], etc., for breast cancer treatment. Table 1 further summarizes several recent advancements in polysaccharide-based NPs and their potential outcome for the treatment of breast cancer.

## 6. Patents

An increasing number of studies have shown that polysaccharide-based NPs hold great potential as effective carriers for delivering anticancer drugs specifically to breast cancer cells. Although CS-based NPs have not yet been used in clinical or commercial cancer treatments, the large number of related patents shows strong efforts to protect new ideas and move this research closer to real-world use. Conversely, while other polysaccharide-based NPs have shown strong preclinical efficacy, the number of patents filed for these systems remains relatively limited. This imbalance points to untapped opportunities for deeper exploration and innovation aimed at addressing current limitations and promoting the clinical advancement of polysaccharide nanocarriers in cancer therapeutics. Table 2 presents a selected compilation of published patents concerning the application of diverse polysaccharide-based NPs in the delivery of anticancer agents for breast cancer treatment.

## 7. Conclusions and Future Perspectives

Polysaccharide-based NPs have emerged as a promising platform for targeted breast cancer therapy, offering a combination of precision and biocompatibility that addresses critical challenges in current treatments. Their inherent biodegradability, hydrophilicity, and functional versatility allow for the creation of sophisticated drug delivery systems capable of enhancing therapeutic efficacy while minimizing systemic toxicity. By utilizing natural materials, such as starch, CS, HA, and alginate, these NPs provide a sustainable and patient-friendly approach to cancer management. One of the key advantages of polysaccharide-based NPs lies in their ability to be functionalized for specific targeting. By attaching ligands or proteins to their surfaces, these NPs can selectively bind to receptors overexpressed on breast cancer cells, ensuring precise drug delivery to the tumor microenvironment. This specificity not only improves the effectiveness of the treatment but also reduces the risk of off-target effects, a major limitation of conventional therapies. Polysaccharide-based NPs further distinguish themselves by their capacity to combat drug resistance, a pervasive challenge in advanced breast cancer cases. Their ability to modulate biological processes such as angiogenesis and immune responses enhances the anti-tumor effects while minimizing the likelihood of disease recurrence. In addition, targeting VEGF receptors with polysaccharide NPs inhibits angiogenesis, a critical process in tumor progression. The ability of polysaccharides to undergo chemical modification further expands their versatility, enabling precise control over characteristics such as solubility, hydrophobicity, and stability. This adaptability is crucial for tailoring NPs to meet the unique demands of breast cancer treatment, such as controlled drug release and enhanced bioavailability under physiological conditions.

Although polysaccharide-based NPs show significant promise for cancer therapy, several barriers must be addressed before clinical translation can be achieved. Despite hundreds of studies demonstrating innovations in their design and preclinical efficacy, clinical investigations remain limited. This gap suggests that excessive complexity and the use of unapproved materials often hinder regulatory approval and raise safety concerns. Therefore, prioritizing simpler, safer, and regulatory-compliant formulations may accelerate clinical advancement. Key priorities include establishing scalable manufacturing processes, ensuring formulation consistency, and meeting regulatory standards for safety and quality. While rodent models have confirmed the anticancer potential and biocompatibility of these systems, studies in larger animal models are essential to evaluate pharmacokinetics (ADME), immune interactions, and potential adverse effects. Such data will be critical to support Phase I clinical trials and eventual clinical adoption. Achieving this goal will require close collaboration between academia, industry, and healthcare institutions, fostering the translation of these nanotherapeutic platforms into mainstream cancer care and offering new hope for breast cancer treatment.

## Figures and Tables

**Table 1:** Recent investigations on various polysaccharide-based NPs and their significant effectiveness for breast cancer treatment.

Type of NPs	Composition of Delivery System	In Vitro Cell Line, In Vivo Model	Key Features/Outcome of the Study	Ref.
Chitosan-based NPs	WZB117/-O-carboxymethyl-chitosan (OCMC) NPs/Metformin (WZB117-OCMC-MET)	MCF-7	➢ NPs possessed 225.67 ± 11.5 nm in size, with PDI (0.113 ± 0.16), and improved %EE (72.78%). ➢ WZB117-OCMC-MET initiates AMPK and suppresses mTOR, indicating growth-inhibitory and apoptotic characteristics. ➢ Enhanced MCF-7 cellular uptake compared to MET alone, further affected cancer cells’ metabolism.	[158]
	DOX/D-α-tocopherol polyethylene glycol 1000 succinate/chitosan (TPGS-g-chitosan) NPs	SK-BR-3; SK-BR-3 induced mouse	➢ Enhanced cellular uptake, cytotoxicity, and bio-adhesion characteristics. ➢ Increased in relative bioavailability of Dox than Docel™ for targeting HER-2 receptor. ➢ Enhanced AUC, MRT, and relative bioavailability achieved via NPs than Docel™. ➢ No significant cytotoxicity in vital organs due to Dox compared to Docel™.	[159]
	Chitosan/poly (γ-glutamic acid) NPs (Ch/γ-PGA NPs) + radiotherapy (RT)	4T1 orthotopic breast tumor mouse	➢ Ch/γ-PGA NPs lessen immunogenicity, and RT diminishes primary tumor burden. ➢ Decreased splenic immunosuppressive myeloid, improved antitumoral CD4 + IFN-γ + population; decreased 4T1 tumor progression and splenomegaly reduction. ➢ Decreased levels of pro-tumor cytokines IL-3, IL-4, IL-10, and CCL4 chemokine.	[160]
	Mertansine (MRT)/cabazitaxel (CBZ)/chitosan (CS)/tetraphenylchlorin (TPC) nanoconjugate	MDA-MB-468 or MDA-MB-231; Mouse model	➢ TPC-CSNPs/MRT/CBZ exhibited higher cytotoxicity compared to free chemotherapeutic molecule. ➢ Strong photodynamic effect induces breast cancer cells; improved biodistribution and accumulation of NPs in major organs.	[161]
	Fucoidan/chitosan NPs/gemcitabine/ErbB-2 antibody (NPs + Gem + Ab)	SKBR3 cells	➢ Increased cellular uptake and targeting improvement via NPs + Gem + Ab (ErbB-2 positive) compared to NPs + Gem + Ab (ErbB-2 negative). ➢ Significant cytotoxicity reported for all the NPs (between 25 and 30%). ➢ Increased (≥80%) cytotoxicity of Gem + Ab system after 24 h compared to free Gem and Gem + NPs.	[162]
	Curcumin/magnetic alginate/chitosan NPs	MDA-MB-231	➢ Significant cytotoxicity in MDA-MB-231 cells compared to HDF cells via Curcumin/magnetic alginate/chitosan NPs. ➢ Higher (3–67 fold) uptake efficiency in cell line via curcumin-loaded NPs than free curcumin.	[163]
Alginate-based NPs	2,3-dimercaptosuccinic acid (DMSA)/Fe_3_O_4_ NPs/chitosan/alginate NPs (CANPs)/doxorubicin (DOX)/hydroxychloroquine (HCQ)	MCF-7, and MDA-MB-231 cell line	➢ Enhanced cytotoxicity and higher cellular uptake of co-administered drugs effectively inhibit the autophagy level of tumor cells.	[164]
	Alginate/chitosan/doxorubicin (DOX) NPs	Murine breast cancer cell line 4T1	➢ NPs (~80 nm) with spherical morphology dispersed in a cyclohexane/dodecylamine organic phase to encapsulate DOX. ➢ MTT assay reported IC50 value (0.15 μg/mL) of NPs after 72 h, higher compared to free DOX (0.13 μg/mL).	[165]
	Alginate NPs/β-pinene/*Ferula gummosa* essential oil	A-375 and MDA-MB-231 cells	➢ NPs exhibited a particle size of 174 to 137 nm, and a surface potential between 12.4 and 28.1 mV. ➢ In vitro study reported higher apoptotic index and breast cancer cells investigated in normoxia and normobaric hyperoxia (NBO) conditions with IC50 values of 76 and 104 µg/mL, respectively. ➢ Bax/Bcl-2 ratio higher, indicating apoptosis and higher sensitivity in A-375 cell line treated with Alginate NPs/β-pinene/*Ferula gummosa* essential oil.	[166]
Hyaluronic acid (HA)-based NPs	Naproxen (NAP)/hyaluronic acid (HA) NPs	MCF-7 breast cancer cells	➢ HA-NAP NPs exhibited a particle size of 300–500 nm via ionic modification. ➢ HA-NAP NPs showed prolonged release of NAP and strong CSC-targeting of MCF-7 cells, with the reduction in cell viability of 45 ± 6%. ➢ Possessed p53-dependent induction of apoptosis via COX-independent pathway and reduction in cell migration (i.e., 76.4% for S-HA-NPs and 61.6% for NAP).	[167]
	HA/polyethyleneimine NPs [HA/PEI NPs])/DTX/α-napthtoflavone	MDR in breast cancer induced by CYP1B1	➢ NPs exhibited a size of 193.6 ± 3.1 nm with spherical morphology. ➢ Downregulation of CYP1B1 expression; further, improvement of DTX bioavailability via HA/PEI NPs.	[168]
	HA/IR780/Doxorubicin (DOX) (HPN)	2D/3D models	➢ Higher encapsulation of IR780 via 2.2 folds, enhanced photothermal and cytocompatibility in a 2D cell uptake model. ➢ Combinatorial applications of IR-HPN and NIR mediated cytotoxicity induction on spheroids in a 3D model. ➢ IR/DOX-HPN + NIR exhibited a significant decrease in cell viability compared to IR/DOX-HPN.	[169]
	Docosahexaenoic acid (DHA)/chlorin e6 (Ce6)/HA [HA-cys-DHA/Ce6 (CHD)]/docetaxel (DTX)	MCF-7 cells	➢ Improved uptake by MCF-7 cell due to CD44 overexpressed via HA. ➢ NPs inhibited microtubule depolymerization and arrested the cell cycle via ROS generation. ➢ DTX/CHD NPs exhibited higher antitumor potential than DTX and CHD NPs under NIR.	[170]
	α1-acid glycoprotein (AGP)/HA NPs (AGP-HA NPs)	MCF-7 cells	➢ Higher migratory tumor suppression (79%) ability by AGP-HA NPs and modulated MDA-MB-231 cells. ➢ Enhanced DOX uptake and reach to cancer cells’ nucleus under incubation via AGP-HA NPs.	[93]
Dextran-based NPs	Dextran/docetaxel (DTX)/docosahexaenoic acid (DHA) (DDD) NPs	MCF-7, and 4T1-tumor-bearing mice	➢ Higher DTX loading capacity in DDD (15.90%) and aqueous solubility of NPs (76.8 ± 5.5 nm). ➢ Improved pharmacokinetic parameters of DDD compared to free DTX, accumulation of tumor in the triple-negative breast cancer model.	[171]
	DTX/docosahexaenoic acid (DHA)/biofunctionalized dextran NPs	MCF-7 cells, Nude mice	➢ NPs exhibited 98.0 ± 6.4 nm and high aqueous solubility, prolonged blood circulation and accumulated majorly in tumor cells compared to normal cells. ➢ Exhibited higher therapeutic effectiveness in both xenograft nude mice models without significant toxicity or systemic side effects, and a reduction in xenografted tumors in MCF-7 induced mice.	[172]
	Curcumin/naringenin/dextran-coated magnetic NPs (CUR-NAR-D-MNPs)	MCF-7 cells	➢ CUR-NAR-D-MNPs exhibited apoptosis and anti-angiogenic effect via ROS. ➢ CUR-NAR-D-MNPs+ radiotherapy induced apoptosis via modulation of P21, P53, higher, CD44, and TNF-α lower, led to cell cycle arrest.	[173]
	Dextran/myristoyl-ECGKRK peptide NPs	MDA-MB-231, and MCF-7	➢ NPs size (248 nm), and zeta potential (10.7 mV) improved myristoyl-ECGKRK peptide loading, showing cytotoxicity (at 50 μm, approx. 85%) in HEK-293 cells but no significant toxicity in breast cancer cells. ➢ Dextran exhibited non-significant cytotoxicity up to 8.3 mg/mL compared to NPs.	[174]
Cellulose-based NPs	MSN/sodium hyaluronate (SH)/SS/oxidized sodium carboxymethyl cellulose (O-CMC) nanohybrids	MCF-7, and MDA-MB-231 cells, Mouse model	➢ Nanohybrids significantly inhibit the tumors without affecting healthy cells via nuclear condensation or fragmentation, supported by in vivo pharmacokinetic assessment.	[175]
	Cellulose Nanocrystals (CNCs) from Chicory plant waste	MCF-7 cells with CYP19	➢ CNCs with 35–37 nm and higher zeta potential exhibited binding affinity of CYP19/Androstenedione-CNCs nanoconjugates. ➢ Overexpression of Bax and p53, decreased mRNA levels of AKT, PI3K, and mTOP levels, leads to a decrease in P-mTOP and PI3Kg-P110 proteins in MCF-7 cells via PI3K/AKT/mTOP signaling pathway after incubation with CNCs at IC50 concentration.	[176]
	Silver NPs (AgNPs)/carboxylated cellulose nanocrystals (Ag-cCNC) from Eucalyptus pulp	MCF-7 cells	➢ AgNPs (16.25 to 21.84 nm) functionalized on the surface of the cCNC, exhibited controlled release (0.02% per day) of Ag+ for 28 days. ➢ Ag-cCNC (200 μg/mL) induces proliferation of MCF-7 cells with 1.01 ± 0.35% cell viability and is non-toxic against healthy cells with 90% viability. ➢ Ag-cCNC demonstrated shear thinning and pseudoplastic fluid behavior.	[177]
	Carnosic acid (CA)/bovine serum albumin (BSA)/chitosan (CH)/cellulose (CL) NPs (CA-BSA-NPs)	MCF-7 and Caco-2 cells	➢ Higher loading capacity of CA and BSA in CA-BSA-NPs and sustained release of 80% in 10 h. ➢ Cell cycle arrest of MCF-7 cells at G2/M (4.73%) in the CA-BSA-NPs treatment. ➢ Upregulation of the GCLC gene and downregulation of the COX-2 gene in cells treated with CA-BSA-NPs compared to untreated cells.	[178]
Starch-based NPs	Curcumin/chitosan/starch/MoS2 nanocomposite	MCF-7 cell	➢ Nanocomposite possessed a 279 nm size with +86.31 mV of zeta potential, 87.25% of entrapment efficiency, and 46.5% of drug loading efficiency. ➢ Apoptosis of MCF-7 was significantly higher in the nanocomposite group compared to the free curcumin suspension, acting in a pH-sensitive manner.	[179]
	Curcumin/polyacrylic acid (PAA)/starch/titanium dioxide (TiO2) nanocomposite	MCF-7 cell line	➢ PAA-Starch-TiO_2_ nanocomposite reported 151 nm of particle size, %EE of curcumin of 87.25% and loading capacity of 49.50%, with higher drug release of curcumin in acidic environment. ➢ Nanocomposite exhibited higher bioavailability and controlled curcumin release compared to free curcumin, leading to improved apoptosis via curcumin-loaded nanocomposite.	[180]
	Starch NP/CG-1521	MCF-7 cells	➢ Significant cytotoxicity of NPs compared with equivalent dose of free CG-1521. ➢ CG-NPs induced cell cycle arrest and apoptosis in MCF-7 cells. ➢ The biological action of encapsulated drug has the similar impact with free drug on gene expression.	[131]
	Chondroitin sulfate-cholesterol (ChS-Chol) nano-assemblies/doxorubicin (Dox)	4T1, MCF-7, and MDA-MB-231 cells	➢ ChS-Chol nanoconjugates dissociate and release DOX in acid pH-responsive system. ➢ Enhanced apoptosis and % of cellular uptake, prevention of cell proliferation compared to free Dox via nanoconjugates.	[181]
	Chondroitin sulfate A (CSA)/chlorin e6 (Ce6)/doxorubicin (DOX) NPs	4T1, and MDA-MB-231 cells, Balb/c mice	➢ NPs exhibited particles of 267 nm, composed of 1.53% of Ce6 and 8.11% of DOX. ➢ CSSC-D and CSSC NPs demonstrated release of DOX or Ce6, enhanced generation of ROS, improving cytotoxic effects in breast cancer cells. ➢ CSSC-D + NIR exhibited tumor growth inhibition in comparison to other groups in vivo.	[182]
	Peptide-grafted chondroitin sulfate A-ss-deoxycholic acid (TCSSD)/DOX TCSSD (TCSSD-D) micelles	MDA-MB-231 cells	➢ TCSSD-D NPs exhibited significant toxicity in glutathione-containing phosphate-buffered solution compared to unmodified DOX in MDA-MB-231 cells. ➢ TCSSD-D micelles demonstrated potent suppression of tumor growth among three DOX-based formulations in triple-negative MDA-MB-231 mice.	[183]
	Indocyanine green (ICG)/calcium-carbonate (ICG@) NP/poly (lactic-co-glycolic acid)-ss-chondroitin sulfate A (PSC) NPs	4T1 cells, and 4T1-bearing Balb/c mice	➢ Spherical NPs with 407 nm particle size of reported pH-sensitive release. ➢ Significant NIR laser irradiation + PSC/ICG@+DOX NPs improved cellular uptake in 4T1 cells. ➢ Enhanced tumor suppression in orthotopic 4T1-bearing mice via PSC/ICG@+DOX NPs; synergistically inhibited the growth of 4T1 cells.	[184]
	d-α-tocopherol polyethylene 1000 glycol succinate (TPGS)/chondroitin sulfate (CS)/paclitaxel (PTX)	MDR breast cancer (MCF-7/MDR) cells	➢ TLA/PTX@CS exhibited 176 nm of size and −18 mV of zeta potential, shown improve the intracellular accumulation of PTX and facilitate the mitochondrial-targeting of lipid-albumin nanosystem. ➢ TLA/PTX@CS entered MDR breast cancer (MCF-7/MDR) cells via CD44 receptor-mediated endocytosis. ➢ Hyaluronidase degraded the CS shell, releasing PTX and halting cell growth. TLA/PTX@CS had longer bloodstream time, resulting in better tumor suppression (75.3%) and significant survival benefit for MCF-7/MDR mice.	[185]
Pullulan-based NPs	Lovastatin (LV)/pullulan (PLV) NPs	MDA-MB-231, MB-453 cells	➢ LV was substituted with varying degrees (DS) with molar ratios of 7.87%, 3.58%, and 3.06% for PLV (1/2), PLV (1/3), and PLV (1/4), respectively. ➢ PLV (1/2) was conjugated to prepare doxorubicin (DXR)-loaded PLV NPs (PLV/DXR NPs) with an average size and zeta potential for PLV (1/2) NPs of 177.6 nm and −11.66 mV, respectively, as determined by dynamic light scattering. ➢ PLV/DXR NPs released DXR sustainably within 72 h. The release was more efficient at pH 5.4 (97.90%) than at pH 7.4 (76.15%). ➢ PLV/DXR NPs inhibited TNBC MDA-MB-231 cells’ growth more effectively than non-TNBC MDA-MB-453 cells. IC50 values: 0.60 μM and 11.05 μM, respectively. More NPs entered MDA-MB-231 cells compared to MDA-MB-453 cells.	[186]
	Biotin/pullulan acetate (Bio-PA) NPs/Epirubicin (EPI)	MCF-7 cells, Nude mice	➢ EPI encapsulated into Bio-PA NPs by dialysis method, and %EE reported 79.8 ± 3.0%. ➢ Higher cellular internalizations of Bio-PA NPs, biotin-mediated endocytosis, were only reported in the biotin-overexpressing HepG2 and MCF-7 cells. ➢ HepG2-bearing nude mice possessed enhanced antitumor effect and distributions of chemotherapeutics in tumor due to Bio-PA NPs loaded EPI.	[187]

**Table 2:** Patents published on polysaccharide-based NPs for breast cancer therapy (Source: Espacenet.com).

Patent No.	Type of NPs	Title of Invention	Year	Ref.
US2022047559A1	Chitosan-based NPs	Drug composition for treating breast cancer and method for manufacturing the same	2022	[188]
CN110339372A	Chitosan-based NPs	Novel RGD-chitosan oligosaccharide silicon oxide/BCSG1-siRNA nanoparticle breast cancer targeted therapy method	2019	[189]
CN108186607A	Chitosan-based NPs	Preparation method of breast cancer targeted chitosan grafted polymer medicine-carrying composite material	2018	[190]
CN108434123A	Chitosan-based NPs	Preparation method of L-peptide modified chitosan drug-loading nanoparticles with breast cancer targeting function	2018	[191]
US12377117B2	Hyaluronic acid-based NPs	Hyaluronic acid nanoparticles comprising NADPH oxidase inhibitors and their uses in treating cancer	2025	[192]
CN117414346A	Hyaluronic acid-based NPs	Metal polyphenol nanoparticle, preparation method and application in preparation of TMEM16A and/or EGFR inhibitor	2024	[193]
WO2018098705A1	Dextran-based NPs	Dextran-magnetic iron oxide nanoparticle, preparation and use in treating cancer and as a contrast	2018	[194]
CN110201181A	Pullulan-based NPs	Pullulan nanoparticles with co-supported lovastatin and doxorubicin, and preparation method thereof	2019	[195]

## Data Availability

No new data were created or analyzed in this study. Data sharing is not applicable to this article.

## Abbreviations

NPs: Nanoparticles; VEGF: Vascular endothelial growth factor; ROS: Reactive oxygen species; SF: Silk fibroin; MDR: Multidrug resistance; P-gp: P-glycoprotein; MTX: Methotrexate; %EE: % encapsulation efficiency; CSNPs: Chitosan nanoparticles; ChoS: Chondroitin sulfate; 5-FU: 5-Fluorouracil; HA: Hyaluronic acid; DOX or Dox: Doxorubicin; CUR or Cur: Curcumin; GO: Graphene oxide; MDRC: Multi-drug resistance cancer; OLA: Olaparib; PTX: Paclitaxel; CMD: Carboxymethyl dextran; DTX: Docetaxel; CSCs: Cancer stem cells; GSH: Glutathione; TNBC: Triple-negative breast cancer.
